# Achieving superelasticity in additively manufactured NiTi in compression without post-process heat treatment

**DOI:** 10.1038/s41598-018-36641-4

**Published:** 2019-01-10

**Authors:** Narges Shayesteh Moghaddam, Soheil Saedi, Amirhesam Amerinatanzi, Alejandro Hinojos, Ali Ramazani, Julia Kundin, Michael J. Mills, Haluk Karaca, Mohammad Elahinia

**Affiliations:** 1 Mechanical & Aerospace Engineering, University of Texas at Arlington, Arlington, TX USA; 2 Department of Systems Engineering, University of Arkansas at Little Rock, Little Rock, AR USA; 3 Mechanical, Industrial, and Manufacturing Engineering Department, The University of Toledo, Toledo, OH USA; 4 Materials Science and Engineering, The Ohio State University, Columbus, OH USA; 5 Department of Mechanical Engineering, MIT, Cambridge, MA USA; 6 ICAMS, Ruhr-University Bochum, Bochum, Germany; 7 Department of Mechanical Engineering, University of Kentucky, Lexington, KY USA

## Abstract

Shape memory alloys (SMAs), such as Nitinol (i.e., NiTi), are of great importance in biomedical and engineering applications due to their unique superelasticity and shape memory properties. In recent years, additive manufacturing (AM) processes have been used to produce complex NiTi components, which provide the ability to tailor microstructure and thus the critical properties of the alloys, such as the superelastic behavior and transformation temperatures (TTs), by selection of processing parameters. In biomedical applications, superelasticity in implants play a critical role since it gives the implants bone-like behavior. In this study, a methodology of improving superelasticity in Ni-rich NiTi components without the need for any kind of post-process heat treatments will be revealed. It will be shown that superelasticity with 5.62% strain recovery and 98% recovery ratio can be observed in Ni-rich NiTi after the sample is processed with 250 W laser power, 1250 mm/s scanning speed, and 80 µm hatch spacing without, any post-process heat treatments. This superelasticity in as-fabricated Ni-rich SLM NiTi was not previously possible in the absence of post-process heat treatments. The findings of this study promise the fast, reliable and inexpensive fabrication of complex shaped superelastic NiTi components for many envisioned applications such as patient-specific biomedical implants.

## Introduction

NiTi-SMAs are well known for their unique properties, i.e., superelasticity and shape memory properties, enabling them to be exploited for functional and smart structures in biomedical and engineering applications^1–3^. These alloys present other beneficial characteristics, such as biocompatibility, wear and corrosion resistance, low modulus of elasticity, and high work output^4–9^. However, the inability to produce complex NiTi parts has limited their potential in a variety of applications, which can be attributed to the difficulties regarding machining of NiTi due to the high reactivity of the alloy, spring back effects, stress-induced martensite, work hardening, and the burr formation^10–12^. Additive manufacturing (AM) is a widely used technique enabling the production of parts with freeform geometry without any tooling which offers a promising alternative to the conventional fabrication routes^13^. Selective laser melting (SLM) is a common powder-bed AM technique which produces metallic components from metallic powder^14^. The combination of NiTi’s superelastic effect, together with freeform design and fabrication in SLM, make it a very attractive combination for biomedical applications^15,16^.

In superelasticity, NiTi can recover a large amount of strain (up to 8% strain) by a reversible stress-induced transformation^15,17–20^. In general, the superelastic response of NiTi extremely relies on the microstructural features of the alloy. The microstructure of AM NiTi has been shown to be different than the conventional NiTi due to the correlation between the melt pools and the associated complex heat transfer, thermal gradients, and grain growth^15^. The leading practice to enhance superelasticity of NiTi-based alloys is precipitation formation through post-process heat treatments (i.e., solution annealing and aging)^21–25^. It has been reported that Ni-rich NiTi alloys with Ni content of more than 50.6 at% are sensitive to heat treatments while it is not practical to perform heat treatments on equi-atomic or Ti-rich NiTi alloys^26^. Depending on the heat treatment conditions (e.g. aging time and temperature) and composition, different types of precipitates are evolved into the sample. In general, the nano-size Ni_4_Ti_3_ precipitates with small relative distance could result in a perfect superelasticity^15^. The AM fabrication parameters have also been shown to strongly affect the microstructure and transformation behavior of AM fabricated NiTi. The important parameters that were found to be influential were laser power (P), scanning speed (v), layer thickness (t), hatch spacing (h), and scanning strategy. Energy input (E) is a combination between these parameters and is defined as “supplied energy via laser beam to a volumetric unites of powder”, which plays a crucial role in microstructure. E, therefore, can be calculated from the following formula (E = P/(v × h × t)).

The main challenge is that the superelasticity, i.e., full strain recovery, in as-cast or as-fabricated conditions is rare to achieve. Up to now, several research groups have focused on the enhancement of superelasticity in AM NiTi-SMAs via post-process heat treatment^15,27–30^. Halani *et al*.^31^ observed stabilized strain recovery of 3% in Laser Engineered Net Shaping (LENS) Ni_55_Ti_45_ (at.%) after solution annealing at 775 °C for 10 h and aging at 500 °C for 1 h. Haberland *et al*.^27^ observed strain recovery of 3.4% with the recovery ratio of 95% in SLM Ni_50.7_Ti_49.3_ (at.%) after solution annealing at 950 °C for 5.5 h and aging at 350 °C for 24 h. Saedi *et al*.^28^ performed solution annealing at 950 °C for 5.5 h and aging at 350 °C for 18 h on SLM Ni_50.8_Ti_49.2_ (at.%); they observed strain recovery of 5.5% with recovery ratio of 95% in the first cycle, and stabilized strain recovery of 4.2% after 10 cycles. In another study, Saedi *et al*.^29^ detected strain recovery of 5.5% in SLM Ni_50.8_Ti_49.2_ (at.%) after aging at 600 °C for 1.5 h, without solution annealing. Post process heat treatments, while proven to be effective, add an additional step which increase the time and costs of product’s preparation. Hence, it is desirable to enhance the superelasticity of AM NiTi-SMAs without the need for post-process heat treatments.

The present study is the first attempt to enhance the superelasticity of SLM NiTi through tuning the microstructure and texture by means of controlling the SLM process parameters. It is proven in literature that a strong columnar texture with preferred [001] direction in single crystals can significantly enhances the superelastic, fatigue, and creep properties of cubic metals such as B2 NiTi^32–35^. Interestingly, [001] texture can also be induced during the SLM fabrication thanks to the directional cooling and layer-by-layer nature of SLM^36,37^. During the SLM fabrication, the well-oriented nucleated {100}_A_ planes grow quickly along the maximum thermal gradients direction and dominate the texture^38^. Providing that the SLM processing parameters act in a way to satisfy “epitaxial growth”, the maximum gradient temperature occurs along the building direction and therefore creates [001]-oriented texture. “Epitaxial growth” is referred to as the fully melting of each layer and partially remelting of the corresponding sublayer, which, in turn, results in the growth of grains along the [001] direction^39,40^. According to Guan *et al*.^41^, h parameter is the most influencing parameter on the microstructure and texture of the alloy, since it directly controls the remelting of the neighboring scan tracks. To this end, the processing parameters of P = 250 W, v = 1250 mm/s, and t = 30 µm were kept constant^28,29,42–45^, where h was altered from 80 µm to 180 µm. In this work, the transformation temperatures (TTs), and hardness of all SLM fabricated samples were evaluated. The superelastic behavior of all the samples was also studied via compressive testing for 10 cycles. Finally, the grain microstructure as well as the texture for various h were evaluated.

## Fabrication and Experimental Procedure

### Fabrication

A slightly Ni-rich Ni_50.8_Ti_49.2_ (at.%) ingot was subjected to an Electrode induction-melting gas atomization (EIGA) by TLS Technik GmbH (Bitterfield, Germany) to produce the NiTi powder (Note: The resultant powders via EIGA technique are spherical and possess low impurity contents^46^). To ensure the layer resolution and flowability, particle size ranging from 25 to 75 µm was used. A SLM machine (Phenix Systems PXM), equipped with a 300 W Ytterbium fiber laser, was used to produce Ni-rich NiTi components. To minimize the level of impurity content within the resultant part, the oxygen level was decreased to 500 ppm prior to fabrication. Impurities of powder and SLM parts are presented in Table 1.

**Table 1:** Impurity contents of the as-received powder and the as-fabricated parts.

Ni_50.8_Ti_49.2_ (at. %)	Oxygen (wt.%) ASTM E1409	Carbon (wt.%) ASTM E1941	Nitrogen (wt.%) ASTM E1477
Powder	250	1250	80
SLM (A1)	250	1250	180

Several cylindrical samples (A1-6) with the diameter of 4.5 mm and length of 10 mm were fabricated. Alternating x/y scanning strategy was implemented for fabrication of the samples. Table 2 summarizes the employed set of processing parameters for each sample.

**Table 2:** List of processing parameters implemented during SLM fabrication.

Sample	Laser Power (P, W)	Scanning Speed (v, mm/s)	Hatch Spacing (h, µm)	Energy Input (E, J/mm^3^)
A1	250	1250	80	83.33
A2	250	1250	100	66.66
A3	250	1250	120	55.55
A4	250	1250	140	47.61
A5	250	1250	160	41.66
A6	250	1250	180	37.03

### Experiment

The SLM fabricated parts were removed from the base plate using electrical discharge machining (EDM). A small portion of the parts (10–40 mg) was cut to determine the Transformation Temperatures (TTs) using a Perkin-Elmer DSC Pyris 1. For optical images, samples were first mounted using epoxy resin and hardener and polished in several stages, using a finer paper and suspensions by a BUEHLER EcoMet/AutoMet 250 Grinder-Polisher. The microstructure was studied by a Keyence VH_Z250R optical microscope. Before optical imaging, samples were etched in a H_2_O (82.7%) + HNO_3_(14.1%) + HF(3.2%) solution for 90 sec. Specimens were further sectioned with wire electric discharge machining and were metallographically prepared with an Allied Multiprep with SiC grinding papers, and polished with felt pads with diamond grit, and Colloidal Silica. Phase analysis was carried out using X-ray diffraction (XRD) in a Bruker D8 X-Ray diffractometer with Cu-Kα radiation fixed with a diffracted beam monochromator. Orientation imaging (OI) was done using an FEI Apreo scanning electron microscope (SEM) at 30 KV with a EDAX Hikari Super EBSD camera. Scanning transmission electron microscopy (STEM) was done with a FEI TF-20 Tecnai 200 kV transmission electron microscope (TEM) adorned with an EDAX Apollo XLT Windowless EDS detector. Samples for the TEM were sectioned from bulk samples via focused ion-beam (FIB) milling. The Vicker hardness of samples was measured by Metal-tester model 900-391D under 100 g loads which was applied for 15 seconds. At least 10 indentations were done to report the average number. Thermo-mechanical tests were conducted by a 100 kN MTS Landmark servo-hydraulic test platform.

## Results

### Microstructural analysis

**Figure 1:**
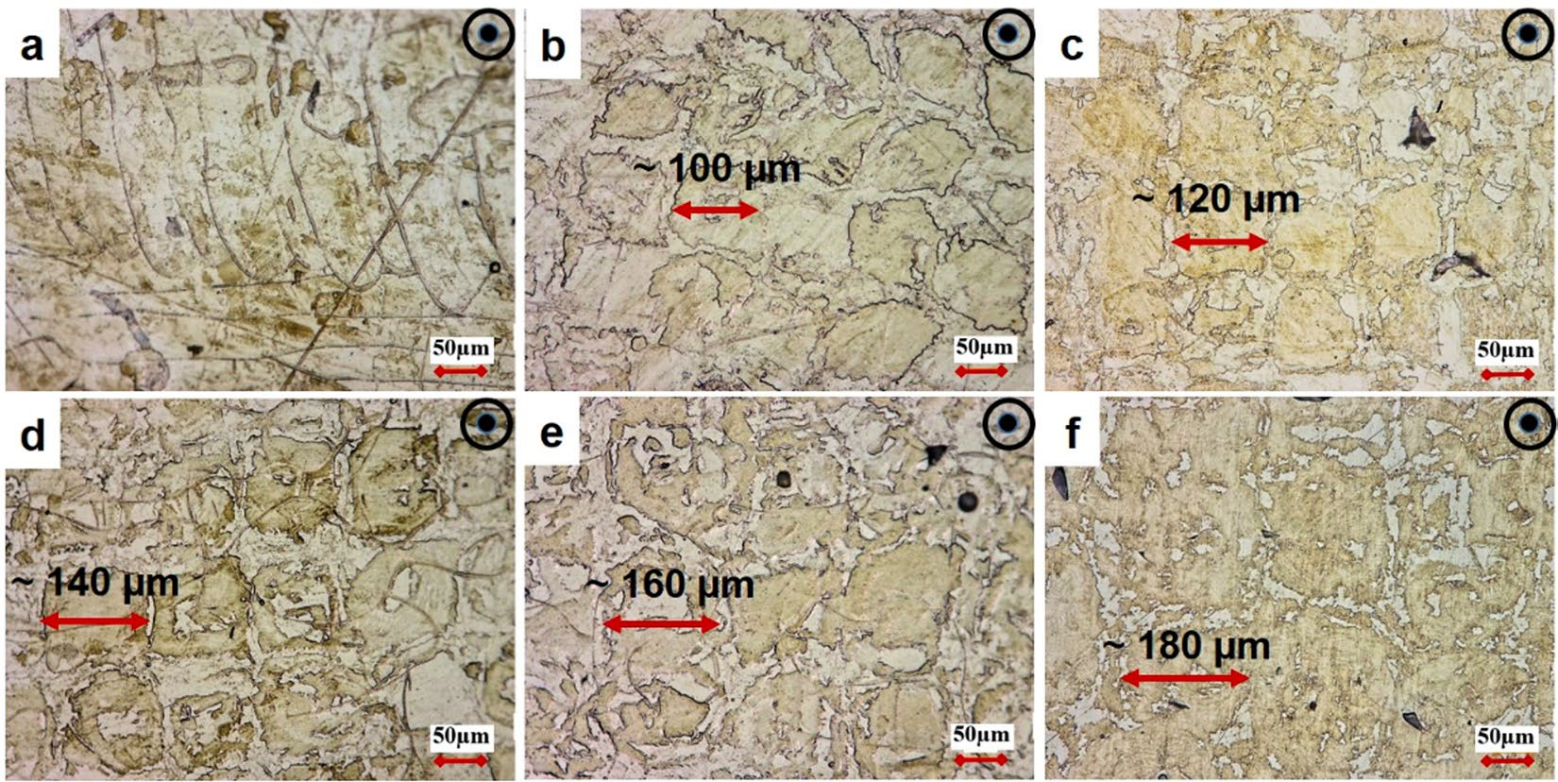
Optical micrographs of Ni_50.8_Ti_49.2_ (at.%) in samples fabricated by different hatch spacing (h) parameter: (**a**) h = 80 µm, E = 83.33 J/mm^3^, (**b**) h = 100 µm, E = 66.66 J/mm^3^, (**c**) h = 120 µm, E = 55.55 J/mm^3^, (**d**) h = 140 µm, E = 47.61 J/mm^3^, (**e**) h = 160 µm, E = 41.66 J/mm^3^, and (**f**) h = 180 µm, E = 37.03 J/mm^3^.

In Fig. 1(a–f), the optical images of SLM NiTi samples processed with different h are compared. The micrographs show that the size of imperfections and the level of porosities increase with h (lower E). Thus, higher h results in a relatively lower density, which can be attributed to a rapid solidification without completely filling the gaps between the melted tracks^47^. For SLM fabrication, it is necessary to implement an optimum high E such that it results in the fully melting of the deposited powder layer as well as the partially re-melting of the previously melted layer ensuring “epitaxial solidification” phenomenon^40^. When h is reduced to 80 µm, the melt pools are observed in the microstructure and the imperfections are eliminated. However, a few microvoids are still detectable which can be attributed to the gases trapped within the melt pools or the Nickel element evaporation^15,47,48^. Finally, overlapped scan tracks are observed in the samples processed with low h, whereas the melt pools become larger and coarser as h increases. The size of the beam diameter (d = 80 µm) might be responsible for the overlapped tracks in h = 80 µm sample (A1), as it results in the remelting of neighboring scan tracks and creates melt pool boundaries similar to welding.

### Phase transformation response and hardness

**Figure 2:**
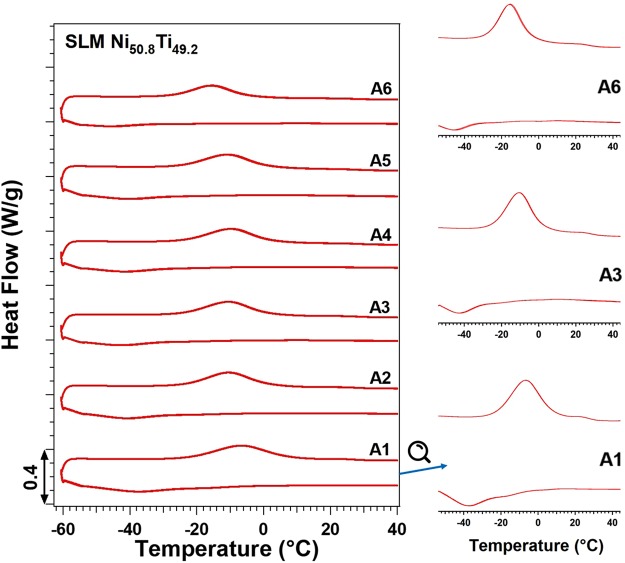
A comparison of DSC results showing the effect of h parameter on TTs of Ni_50.8_Ti_49.2_ (at. %).

In Fig. 2, the DSC curves of samples processed with selected h are illustrated. Both forward and back transformation peaks are broad; hence, magnified curves of A1, A3, and A6 samples are also shown. While the martensitic transformation is completed through multiples steps, the austenite peak is relatively sharper and accompanied with a shoulder. It is noteworthy that the last martensite peak becomes sharper as h decreases suggesting less heterogeneous microstructure. Such broad and multi-step transformation may indicate the presence of ultra-fine coherent precipitates or impurities, producing a strong resistance to the large lattice deformation associated with B19′. Such precipitates/impurities, if available, also can cause inhomogeneous Ni distribution and matrix composition^49^.

**Figure 3:**
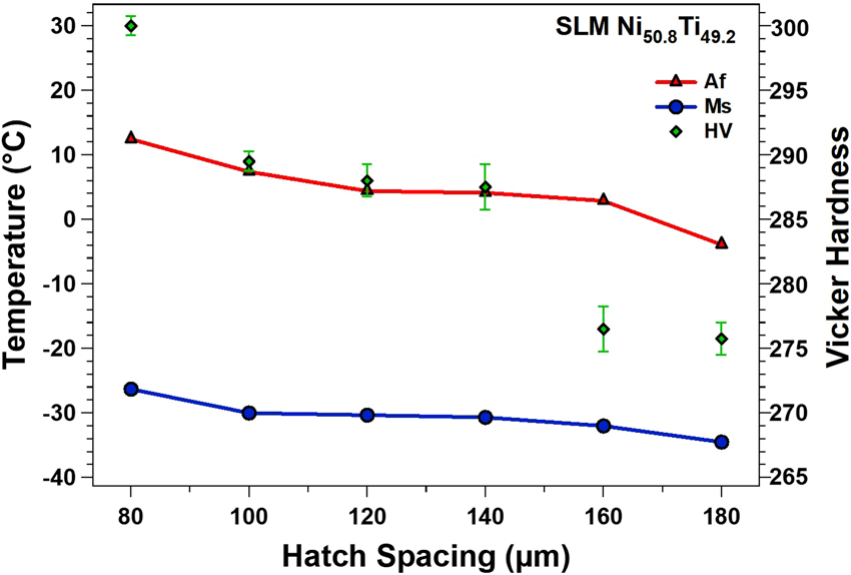
Transformation temperatures (TTs) and Vicker hardness of SLM Ni_50.2_Ti_40.8_ (at. %) samples as a function of (a) h and (b) v.

The TTs are extracted from the DSC graphs and their variation as a function of E is given in Fig. 3. The M_s_ temperature has been extracted from the last and sharpest peak of the martensitic transformation. Additionally, Vicker hardness values of the samples have been included. It is clear that TTs are increased as the parameter h is decreased (higher E). The corresponding DSC plots are also plotted in Fig. 2(b). In the circumstances where the applied E is higher, the melt pools are held at a relatively higher temperature, and therefore, the matrix composition shifts to a higher Ti content due to the higher rate of Ni evaporation (note: the melting point of Ni element is lower than that of Ti). It should be noted that higher E is also associated with a higher level of impurity, the formation of Ti-rich impurities, and hence the Ti depletion in the NiTi matrix. However, it is reported that the Ni depletion associated with Ni evaporation compensates or even overcomes the Ti depletion associated with the formation of Ti-rich impurities^15,50^. Thus, the corresponding Ni depletion results in an increase in the TTs^15^. Finally, an increasing trend for Vicker hardness of samples is also observed as h is decreased (higher E).

### Superelastic response

**Figure 4:**
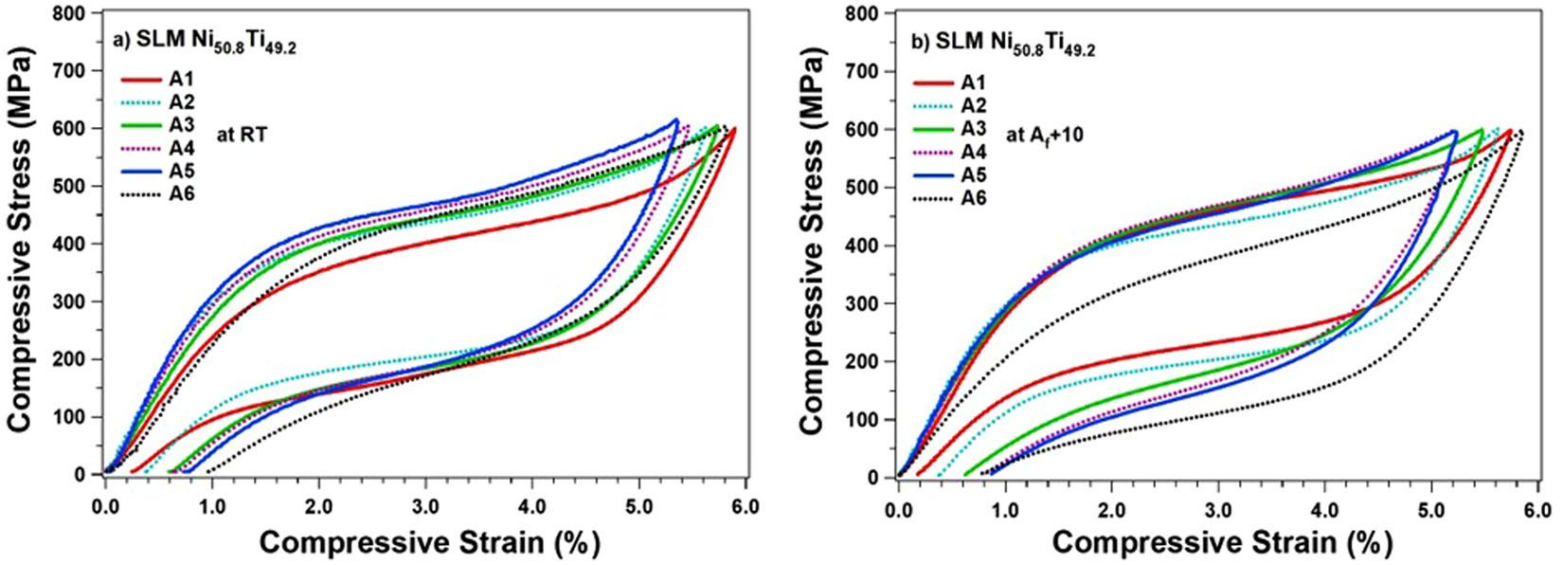
The superelastic response of samples A1–6 tested at: (**a**) room, and (**b**) A_f_ + 10 temperatures.

In Fig. 4(a), the superelastic responses of samples processed with different h are investigated at room temperature (RT). All samples were first loaded up to 600 MPa, to ensure the loading plateau has reached the “apparent” elastic regime of the martensite phase, and then were unloaded. The plots indicate that the lowest h sample (80 µm) results in the best superelastic response at room temperature (A1). However, to be able to compare all the important characteristics of the superelastic response (e.g., critical stress for martensitic transformation, strain recovery, and recovery ratio), the superelastic tests were also conducted at A_f_ + 10 °C and were plotted in Fig. 4(b). Again, the A1 showed the best superelastic response and the highest strain recovery of 5.62% with recovery ratio of 98% at A_f_ + 10 °C. However, these two characteristics are degraded as h is increased. The poorest response belongs to the sample processed with h = 160 µm, with 4.35% strain recovery and 83% recovery ratio (A5). Further, the plots present no considerable difference in terms of critical stresses for the samples processed with h up to 140 µm, however, a drastic drop was observed in the sample fabricated with highest h (180 µm) (A6). This can be explained by the fact that the neighboring scan tracks are not fully bonded and there exists gaps and porosity along the melt pool boundaries^51^.

**Figure 5:**
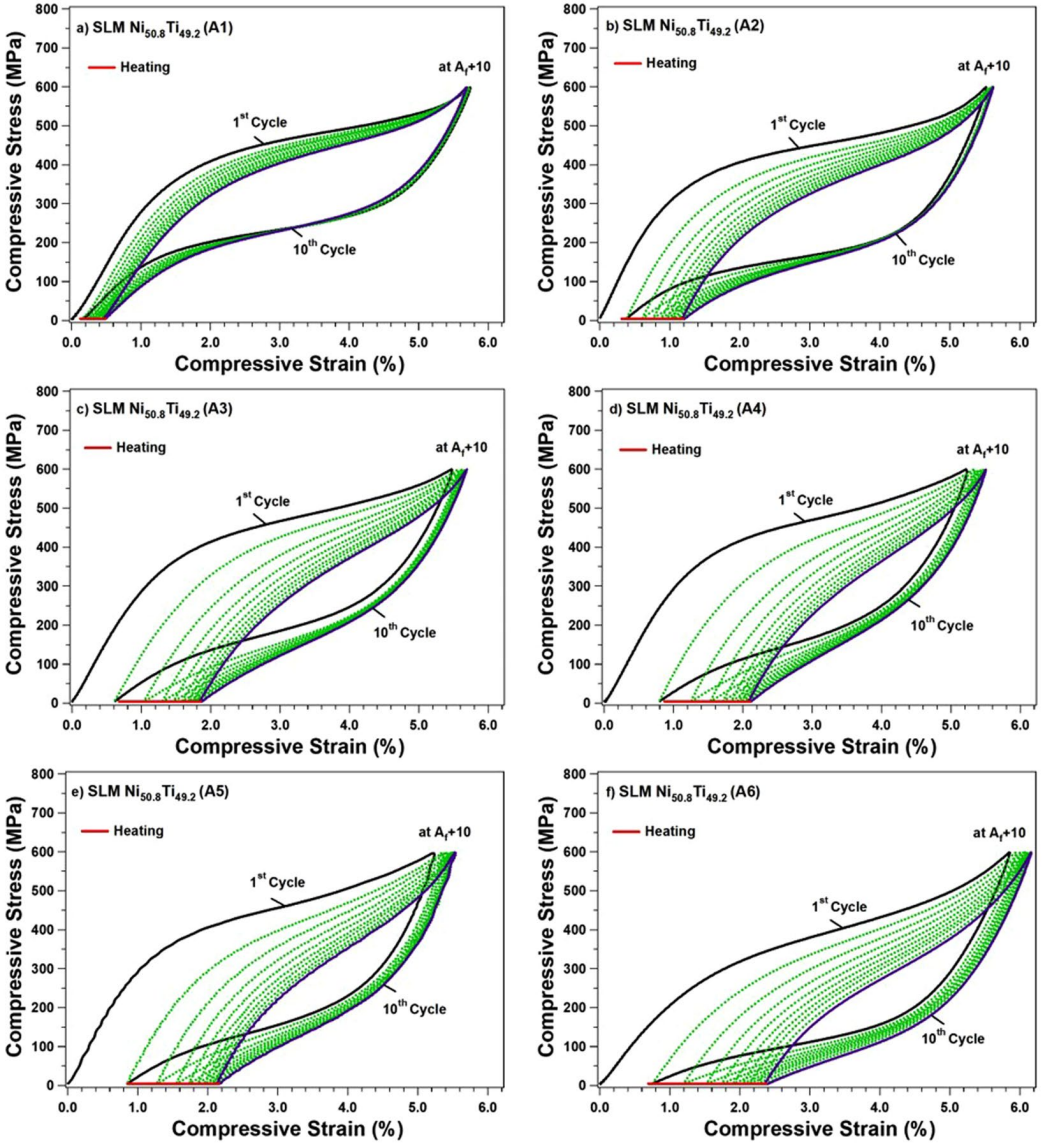
Superelastic cycling of SLM Ni_50.8_Ti_49.2_ (at. %) fabricated by different h parameter: (**a**) h = 80 µm, (**b**) h = 100 µm, (**c**) h = 120 µm, (**d**) h = 140 µm, (**e**) h = 160 µm, and (**f**) h = 180 µm.

In Fig. 5(a–f), cyclic responses of the samples processed with different h are presented at A_f_ + 10 °C, respectively, to analyze the stability of each sample. The irrecoverable strain (ε_irrec_), recoverable strain (ε_rec_), and total strain (ε_tot_.) of the first and last cycles are also summarized in Table 3. As expected, the most stabilized superelastic response is observed for the sample processed with the lowest h while the hysteresis is degraded as h is increased. The highest strain recovery of 5.2% is attributed to the sample fabricated by h = 80 µm. As h is increased, the stabilized strain recovy is degraded where the poorest strain recovery of 3.4% is attributed to the sample fabricated by h = 160 µm. It should be noted here that there was a slight increase in the recovery ratio, ε_rec_, and the ε_tot_ in the h = 180 μm sample, reasons for this are still not understood and are still being explored.

**Table 3:** Summary of the cyclic response of SLM Ni_50.8_Ti_49.2_ (at. %) samples at first and last cycle.

SLM Ni_50.8_Ti_49.2_ (at. %)	Applied Stress (MPa)	1^st^ Cycle			10^th^ Cycle		
		ε_tot_ (%)	ε_Irrec_ (%)	ε_rec_(%)	Recovery Ratio (%)	Total ε_Irrec_ (%)	Stabilized ε_rec_ (%)
A1	600	5.72	0.10	5.62	98	0.47	5.20
A2	600	5.51	0.38	5.13	93	1.21	4.44
A3	600	5.47	0.62	4.85	89	1.86	3.84
A4	600	5.12	0.8	4.32	84	2.09	3.41
A5	600	5.22	0.87	4.35	83	2.15	3.40
A6	600	5.85	0.77	5.08	87	2.37	3.79

## Discussion

### Selection of SLM processing parameters in favor of superelasticity

It was known from the literature of SLM NiTi alloys that post-process heat treatments (i.e., solution annealing and aging) were required to improve superelasticity in the as-fabricated alloy^15,27–30^. Hence, finding a way to improve superelasticity and stability in the as-fabricated SLM NiTi was critical to avoid the extra costs and efforts associated with post-process heat treatments. The approach in this research was to enhance superelasticity through altering h parameter, while implementing a high P (250 W) to ensure crack-free microstructure. It was revealed that the employment of low h (80 µm) and moderate E (83.3 J/mm^3^) in sample A1 resulted in the highest stabilized strain recovery of 5.2% at room and body temperature.

Several factors could account for the improved superelasticity, including but not limited to the development of a preferred texture, presence of precipitates, or grain refinement. To illustrate these claims and quantify the superelasticity phenomenon, EBSD, XRD, and TEM measurements were performed on the selected as-fabricated SLM NiTi Ni_50.8_Ti_49.2_ (at. %) samples.

### Origin of superelasticity in as-fabricated SLM NiTi

#### XRD analysis

**Figure 6:**
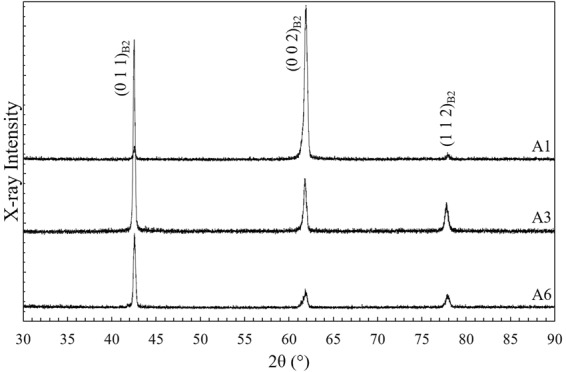
XRD measurements of the A1 (h = 80 μm), A3 (v = 1250 mm/s), and A6 (h = 180 μm) fabrications.

In Fig. 6, XRD spectra of the h = 80 μm (A1), h = 120 µm (A3), and h = 180 μm (A6) fabrications is shown. The spectra of the fabrications only exhibit peaks associated with the austenite B2 phase, as indicated by (011)_B2_, (002)_B2_, and (112)_B2_ reflections. The presence of strong B2 peaks confirms that the majority volume fraction of the samples are B2 phase. This agrees with the findings from DSC, where single-step transformation from B2 austenite to monoclinic B19′ martensite is observed. Apparent noise and broadening at the base of the XRD peaks could have been a remnant from secondary phases with a low volume fraction. Variation in the peak intensities is likely to be due to the significant difference in the grain orientations due to different deposition conditions, as will now be discussed.

#### EBSD analysis

**Figure 7:**
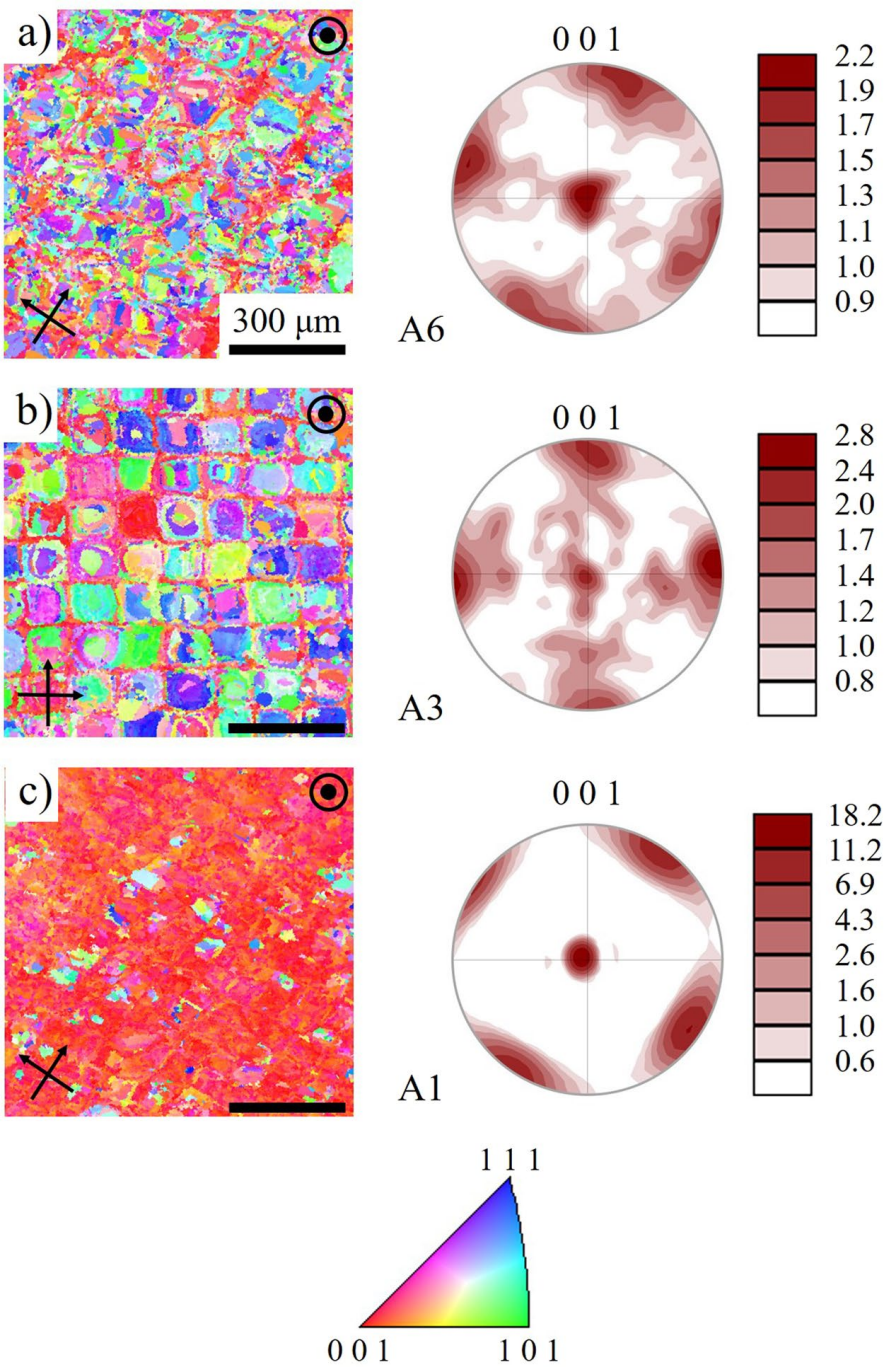
The inverse pole figure maps (left) and the pole figure texture plots (right) of the (**a**) h = 80 μm (A1), (**b**) h = 120 μm (A3), and (**c**) h = 180 μm (A6) fabrications. The micrographs are oriented along the build direction and the scanning directions in each case are indicated by the black arrows. All micrographs have 300 μm scale bars.

In Fig. 7(a–c), the OIM inverse pole figure (IPF) images with respective pole figures are demonstrated for the h = 80 µm, h = 120 µm, and h = 180 µm specimens (A1, A3, and A6), respectively. The pole figures in each case are relative to the build direction. From the pole figures the specimens with a h greater than 80 μm (e.g., A3, A6), show a weak [001] texture, whereas the h = 80 μm specimen shows a strong [001] texture. The IPF image of h = 180 μm specimen (A6) shows an irregular coarse grain structure. There is an apparent orientation preference along [001] in the narrow channels that form along the edges of the melt pool. The distance between the channels is consistent with h. Meanwhile, the “island” regions between the channels are typically comprised of multiple grains which do not reveal any apparent preferential orientation. For the h = 120 μm sample, A3, there is a similar channel grid with a [001] texture, once again consistent with h. However, the islands consist of similar grain orientations, or mix of irregular fine grains. The h = 80 μm specimen (A1) does not exhibit a pronounced channel and island structure. A strongly pronounced texture along the [001] was also observed. From these pole figures it is also apparent that [100] and [010] directions are aligned with the laser scanning directions. Moreover, as it was shown in Fig. 3, the Vicker hardness of samples was increased as the h decreased (higher E) and thus the highest HV was observed for A1. The higher hardness when a higher E is implemented can be related to the grain refinement and texture along the [001] direction, which, in turn, yields to denser microstructure, higher strength, and hardness. The pronounced [001] texture and higher hardness partially explains the high recovery ratio for the h = 80 μm fabrication^52,53^. The development of the [001] texture could technically improve the superelasticity in compression, because the Schmid factor for the slip systems for [001] orientation in B2 phase NiTi (i.e., <001>{110} and <001>{100}) is zero under compressive loading^33,53^. A relatively low Schmid factor means that the critical stress for slip is much higher than the critical stress for stress induced martensite transformation (SIMT), thereby minimizing plastic deformation and improving the superelasticity^53^. It should be noted that different slip systems can be activated depending on the single crystal orientation and stress state (tension/compression)^54^. Superelastic response along other orientations (e.g., transverse to the build direction) would be less pronounced due to the weaker texture and unfavorable orientations^38,55^. The precipitation hardening is another important factor that could also result in enhanced superelastic effects which was further explored through TEM analysis.

#### TEM analysis

**Figure 8:**
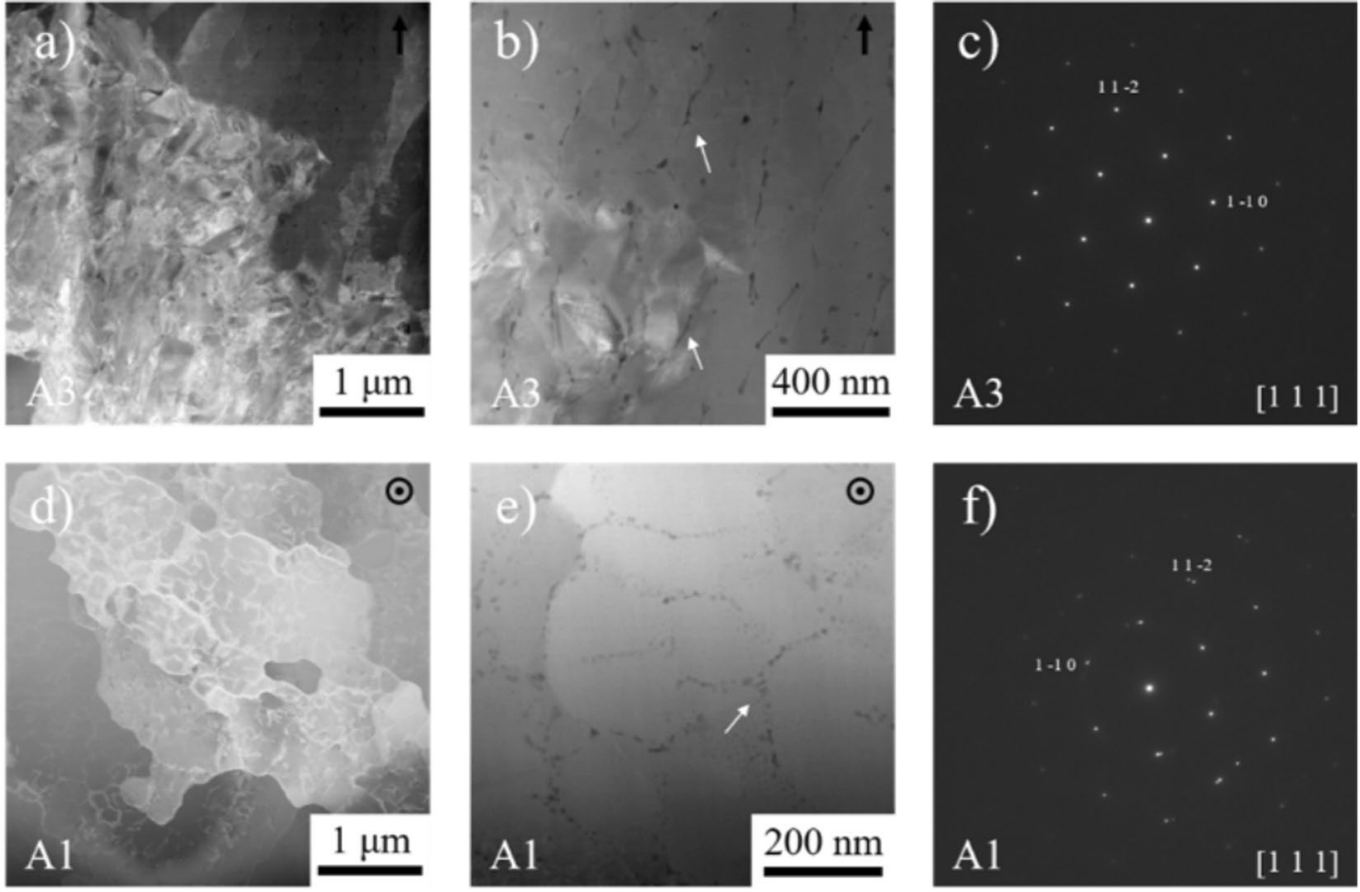
STEM high angle annular dark field (HAADF) micrographs (**a**,**b**,**d**,**e**) and SADPs (**c**,**d**) of the h = 120 μm (A3) channel (**a**–**c**) and h = 80 μm (A1) (**d**,**e**) fabrications are shown here. The white arrows in (**b**) point to the Ti and O enriched areas in the A3. The white arrows in (**e**) show the phase that adorns the low angle boundaries that are in O and Ti in the A1. The SADPs in (**c**) and (**f**) are aligned with the [111] zone axis in the B2 structure.

TEM foils were extracted from the h = 80 μm (A1) and h = 120 μm (A3) samples, represent the two extremes of the deposition conditions in the study. The h = 80 μm TEM foil was taken transverse to the build direction and was oriented at 45° angle from the build direction within the melt pool. The A3 foils were taken parallel to the build direction and were plucked from the island and channel region. STEM micrographs of the h = 80 μm (A1) and h = 120 μm (A3) fabricated specimens can be seen in Fig. 8. For the h = 120 μm specimens large regions dislocations arrays were observed throughout the structure as seen in Fig. 8(a). In Fig. 8(b) stringer like secondary phases were present within the boundaries, EDS further revealed enriched O and Ti as well. In addition, there were some particles that revealed some enrichment in C. In similar studies by Ma *et al*.^56^, O-rich precipitates, most likely Ti_4_Ni_2_O precursors, were found in similar regions. Observations of the h = 80 μm specimen, in Fig. 8(d), revealed networks of low angle boundaries (LAB), which are most likely a remnant from the solidification process. Similar LAB structures have been observed previously in SLM fabricated NiTi and 316 stainless steel^56,57^. In Fig. 8(e), the presence of finer secondary phases with the average size of ~80 μm and the standard deviation of ~6 nm within LABs were observed. Further EDS analysis of these secondary phases revealed enrichment in O and Ti. The enrichment in the Ti and O has been shown previously to represent the formation of Ti_4_Ni_2_O^58^. Similar precipitates have been seen by Sam *et al*.^59^ but were not directly attributed to be affecting the superelastic response. The presence of these “oxides”, at larger sizes (>1 μm), have been observed previously to lower the M_s_ temperature, but, the ultimate effect on the superelastic response was negligible. These “oxides” that are substantially larger will tend to act as crack initiators when adjacent to voids in structural fatigue^60^.

Selected area diffraction patterns (SADPs) on [111] type zone axes from both specimens, seen in Fig. 8c,f, were collected, in an effort to detect the presence of Ni_4_Ti_3_ superlattice reflections^61^. The lack of superlattice reflections in the diffraction patterns in Fig. 8c and f proves the absence of Ni_4_Ti_3_ precipitates in both the h = 80 μm and h = 120 μm fabrications. Ma *et al*.^56^ had previously shown in that at a smaller h value, small Ni-rich precipitates had formed at a fine scale (1–3 nm) due to the increased heat from thermal cycling at small h. The presence of these fine precipitates were understood to be draining the surrounding matrix of excess Ni and thus raising the martensitic transformation temperature^56^.

In summary, there exists an unmet need for a methodology to enhance the superelasticity of as-fabricated SLM NiTi, without the need for post-process heat treatment. The as-fabricated specimen processed with said parameters demonstrated remarkable superelasticity behavior, i.e., strain recovery of 5.62% with recovery ratio of 98% in the first cycle, and the stabilized strain recovery of 5.2% after 10 cycles. Such improved superelasticity (under compression) is attributed to the strong texture along the [001] direction, which is typically observed within [001]-oriented single crystal conventional NiTi alloys^62^. While this level of superelasticity under compressive loading is of great interest, a next critical step is to evaluate the superelasticity of the as-fabricated Ni-rich NiTi alloy under tension. In a recently published paper^63^, however, it has been shown that the SLM NiTi alloy presents premature failure due to the presence of numerous un-melted powders concentrated in the edges of the specimens, which could act as crack initiation sites. Should this problem be successfully addressed, a higher level of recoverable strain under tension is expected, probably 1.5 times larger due to the differences in deformation mechanisms with loading direction and the unidirectional nature of twin deformation^64–68^.

In addition to the texture, precipitation also plays an important role in enhancing superelasticity. In SLM process, the previously deposited material is thermally cycled from the repeated laser passes, which might result in the precipitation of Ni_4_Ti_3_ particles in the Ni-rich NiTi alloy. Ma *et al*.^56^ had done simulations to best capture the complex thermal history of the deposited material in addition to simulating and predicting the precipitation behavior of the Ni_4_Ti_3_ precipitates. Their simulations indicated that precipitates would be most probable to form at small h and high E; however, the presence of the Ni_4_Ti_3_ precipitates was not confirmed in their work. It should be noted that in their study, a substantially lower laser power (50 W) was used. The broadened DSC peak could indicate the presence of very fine precipitates (or nuclei of precipitates). According to Sehitoglu *et al*.^53^, even for the [001]-oriented single crystals, the good superelasticity can only be obtained after aging treatment. However, one should note that Kaya *et al*.^69^ and Liu *et al*.^70^ observed superelasticity in the [001]-oriented Ni_51_Ti_49_ (at. %) and Cu_71_Al_18_Mn_11_ (at. %) alloys in unaged conditions, respectively. While superelasticity can be achieved in low h samples, it is necessary to perform post-process heat treatment on high h samples to achieve superelasticity.

## Conclusion

In this study, we present a method to tailor the superelastic response by adjusting the processing parameters for SLM fabrication. It has been shown that moderate E through lower h results in better superelasticity behavior within as-fabricated SLM Ni_50.8_Ti_49.2_ (at. %). The main findings of the study are outlined as follow:

- Hatch spacing is a very effective parameter to tailor the grain size, shape and orientation, and thus the superelastic response of SLM fabricated NiTi alloys.
- As h was decreased from 180 to 80 µm, clearer melt pool boundaries were observed in the SLM NiTi samples. The lowest h (80 µm) resulted in the finest melt pools due to the intense overlapping between the neighboring scan tracks.
- At lower h, higher TTs and Vicker hardness were observed in the SLM NiTi samples due to the grain refinement in the NiTi matrix.
- Without any heat treatment, the as-fabricated samples with low h (80 µm) demonstrated remarkable superelastic response with strain recovery of 5.62% and recovery ratio of 98% in the first cycle. The stabilized strain recovery was 5.2% after 10 cycles.
- The enhanced superelasticity of SLM NiTi fabricated by h = 80 µm in as-fabricated condition can mainly be attributed to the strong texture along the [001] orientation along the building direction, as the TEM images confirmed the absence of Ni_4_Ti_3_ precipitates.
